# Selective entrainment of the *Drosophila *circadian clock to daily gradients in environmental temperature

**DOI:** 10.1186/1741-7007-7-49

**Published:** 2009-08-11

**Authors:** Jake Currie, Tadahiro Goda, Herman Wijnen

**Affiliations:** 1 University of Virginia, Department of Biology, Charlottesville, VA, USA

## Abstract

**Background:**

Circadian clocks are internal daily time keeping mechanisms that allow organisms to anticipate daily changes in their environment and to organize their behavior and physiology in a coherent schedule. Although circadian clocks use temperature compensation mechanisms to maintain the same pace over a range of temperatures, they are also capable of synchronizing to daily temperature cycles. This study identifies key properties of this process.

**Results:**

Gradually ramping daily temperature cycles are shown here to synchronize behavioral and molecular daily rhythms in *Drosophila *with a remarkable efficiency. Entrainment to daily temperature gradients of amplitudes as low as 4°C persisted even in the context of environmental profiles that also included continuous gradual increases or decreases in absolute temperature. To determine which elements of daily temperature gradients acted as the key determinants of circadian activity phase, comparative analyses of daily temperature gradients with different wave forms were performed. The phases of ascending and descending temperature acted together as key determinants of entrained circadian phase. In addition, circadian phase was found to be modulated by the relative temperature of release into free running conditions. Release at or close to the trough temperature of entrainment consistently resulted in phase advances. Re-entrainment to daily temperature gradients after large phase shifts occurred relatively slowly and required several cycles, allowing flies to selectively respond to periodic rather than anecdotal signals. The temperature-entrained phase relationship between clock gene expression rhythms and locomotor activity rhythms strongly resembled that previously observed for light entrainment. Moreover, daily temperature gradient and light/dark entrainment reinforced each other if the phases of ascending and descending temperature were in their natural alignment with the light and dark phases, respectively.

**Conclusion:**

The present study systematically examined the entrainment of clock-controlled behavior to daily environmental temperature gradients. As a result, a number of key properties of circadian temperature entrainment were identified. Collectively, these properties represent a circadian temperature entrainment mechanism that is optimized in its ability to detect the time-of-day information encoded in natural environmental temperature profiles. The molecular events synchronized to the daily phases of ascending and descending temperature are expected to play an important role in the mechanism of circadian entrainment to daily temperature cycles.

## Background

Due to the rotation of our planet around its own axis most life forms on earth are exposed to daily rhythms in environmental light and temperature. In response, internal daily biological timekeepers, termed circadian clocks, have evolved. These circadian clocks provide organisms with the ability to reliably predict regular daily changes in their environment and to organize their bodily functions and behavior in a coherent daily schedule [1-6]. The selective advantage of possessing a circadian clock that is tuned to environmental rhythms has been directly demonstrated by experiments using cyanobacteria [7] and *Arabidopsis *[8,9]. The defining properties of circadian clocks include (1) ~24-h (circadian) periodicity, (2) autonomous time keeping under constant conditions, (3) entrainment to environmental time cues such as light or temperature, (4) control of overt biological rhythms, and (5) maintenance of a constant pace over a range of environmental temperatures [1-6]. The molecular circadian clocks that have been described in higher eukaryotes all have transcriptional feedback circuits that allow them to create self-sustaining molecular oscillations in gene expression. In the *Drosophila *clock the positive transcriptional regulator CLOCK/CYCLE (CLK/CYC) induces peak transcript levels of a number of clock-controlled genes, including *period *(*per*), *timeless *(*tim*), *vrille *(*vri*) and *PAR-domain protein 1 *(*Pdp1*). Subsequent accumulation and nuclear entry of PER and TIM proteins results in direct inhibition of CLK/CYC, whereas VRI acts as a negative transcriptional regulator for the *Clk *promoter and PDP1 functions as a transcriptional activator. Elaborate post-translational mechanisms are at work to ensure that gene expression oscillations are produced with a stable circadian period length.

While the molecular mechanisms underlying circadian time keeping and synchronization to environmental light/dark cycles are relatively well understood, this is not yet the case for temperature entrainment. Circadian clocks have a complex relationship with environmental temperature, showing the ability to synchronize to daily temperature cycles as well as adjust their daily phase to seasonal differences in average temperature while retaining the ability to run at the same speed over a wide range of average daily temperatures [1,4,10,11]. Repeated daily temperature cycles can entrain clocks, while single temperature cycles, pulses, or steps can reset circadian phase.

Prior to the present study, it was, however, not known what specific features of daily temperature profiles determined circadian phase and how those phase determinants were interpreted in the context of complex environmental profiles that better resemble natural conditions. The analyses outlined below methodically addressed these issues and indicated that the *Drosophila *circadian clock is remarkably sensitive to the environmental daily time cues represented in natural environmental temperature profiles.

## Results and discussion

### Circadian behavior is efficiently synchronized by daily temperature gradients

**Figure 1:**
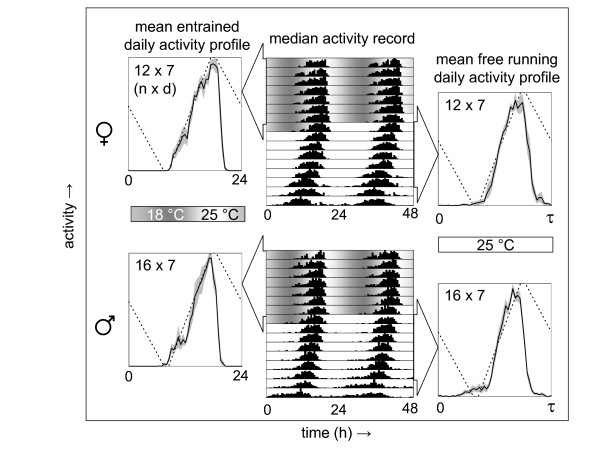
**Efficient entrainment of circadian behavior to daily temperature gradients**. Twelve female and sixteen male Canton-S flies were analyzed individually for synchronization of locomotor activity during and after exposure to a daily temperature gradient in constant darkness. The top and bottom center panels are double-plotted actograms in which the median locomotor activity per half-hour interval (black vertical bars) is plotted. Each row represents a 2-day interval (starting at 12 am), of which the second day is repeated as the first day on the next row. The activity records start with 7 days of exposure to a temperature cycle that gradually ramps between a 1-h interval at 18°C and a 1-h interval at 25°C. On the eighth day the temperature is held at 25°C and this condition is maintained thereafter (see profile Z5C4 in Additional file 2). The panels on the left and right represent the mean (solid line) ± Standard Error of the Mean (S.E.M.; shading) daily activity profiles for the indicated 7-day intervals. The left-hand panels correspond to the 24-h activity profile in the presence of the temperature gradient, whereas the right-hand panels correspond to the activity profile associated with the period length (*τ*; here ~23.5 h) measured during the subsequent free run at constant 25°C. The shading in the top part of the actograms and the dotted lines in the activity profiles represent the daily temperature gradient. The numbers of individual flies (*n*) and experimental days (*d*) contributing to each activity profile are indicated.

To determine whether the *Drosophila *clock could be synchronized to daily cycles of gradual temperature changes circadian entrainment of locomotor behavior was examined during and after exposure to daily temperature gradients in constant dark (DD) conditions (see Additional file 1). An example of the strong synchronizing effect of daily temperature gradients is represented in Figure 1. Flies were exposed to a gradual 18°C to 25°C temperature cycle and then released at constant 25°C. The maximal rate of temperature change during entrainment was only ~0.8°C/h, yet the phase of locomotor activity for both male and female flies was efficiently synchronized to coincide with the time of day corresponding to the temperature maximum. This phase relationship persisted during free running conditions indicating that the synchronized behavior represented a synchronized circadian clock rather than a purely temperature-driven direct effect on behavior.

### Daily peak locomotor activity synchronizes to peak temperature in a daily temperature gradient

**Figure 2:**
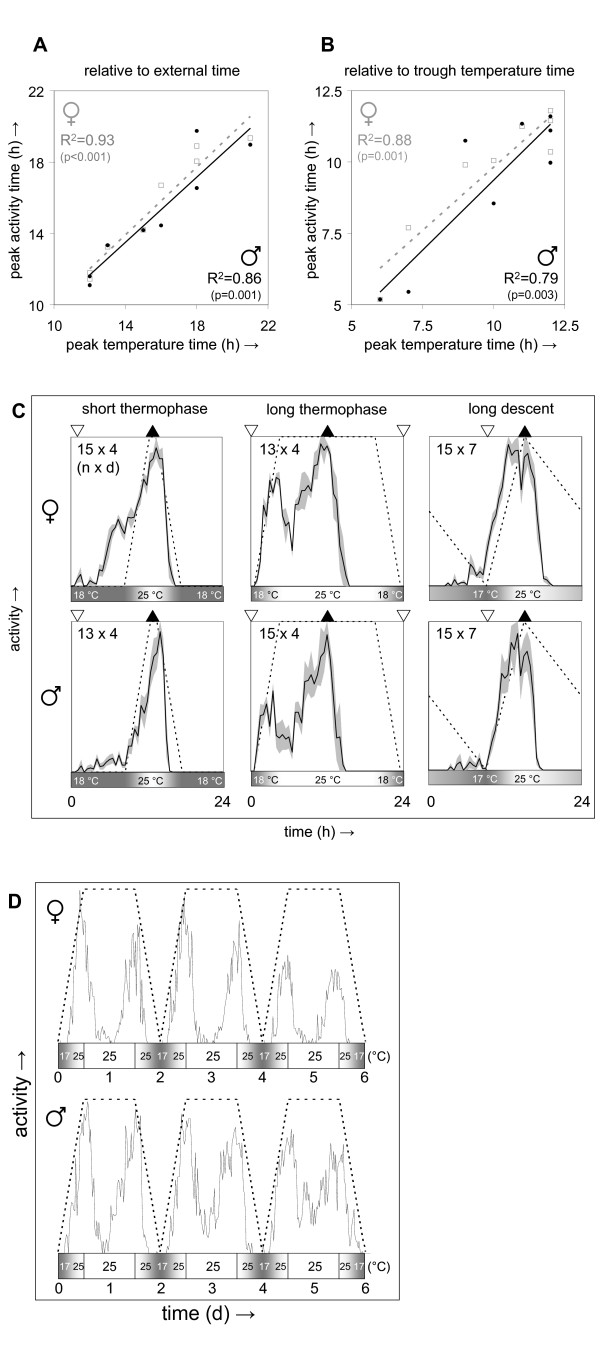
**Daily peak temperature is tracked by daily peak locomotor activity**. Locomotor activity was recorded for Canton-S flies subjected to one of eight different daily temperature gradients in constant darkness (Z1-8 as indicated in Additional file 2 and Additional file 3). The time of entrained daily peak activity was determined from median activity records and plotted as a function of the time associated with the middle of the peak temperature phase either relative to external time (Eastern Standard Time; panel **A**) or relative to the time of the middle of the trough temperature phase (panel **B**). The data is plotted separately for each gender along with trend lines and associated correlation coefficients and their significance (2-tailed). Examples of entrained daily activity profiles are represented in panel **(C)**. In a separate experiment Canton-S flies were exposed to a temperature protocol that violates the normal arrangement of the subjective daily phases of temperature ascent and descent relative to the midpoint of the temperature maximum. Median 6-day activity profiles representing groups of 8 males and 8 females synchronized to this protocol are shown in panel **(D)**. The dotted lines in the activity profiles as well as the shaded bars underneath for panels (C) and (D) represent the daily temperature gradient, while the closed and open arrowheads at the top of the diagrams in panel (C) correspond to the middle of the peak and trough temperature phase, respectively. The numbers of individual flies (*n*) and experimental days (*d*) are indicated in panel (C).

Analyses of temperature-entrained locomotor behavior such as those shown in Figure 1 suggested that the entrained activity peak phase was determined by the phase of the temperature maximum. This hypothesis was systematically tested by comparing the peak phase of synchronized locomotor activity with that of the entraining daily temperature gradient for eight different temperature protocols varying in amplitude (between 7°C and 12°C), peak phase, absolute temperature (from between 14°C and 22.5°C to between 22°C and 30°C), relative duration of thermophase, cryophase, temperature ascent or descent (see Z1-8 in Additional file 2 and Additional file 3). These protocols represent a diverse set of daily temperature profiles in which the duration of the temperature peak and trough vary from a discrete point in time to 15 h and the phases of ascending and descending temperature may be as short as 4 h or as long as 12 to 15 h. The results of this analysis are summarized in Figure 2A and 2B. Most of the variance in peak activity phase is explained by a linear relationship with the time of peak temperature (see Figure 2A and 2B, and Additional file 3). Moreover, this relationship is relatively insensitive to changes in the time of trough temperature or other features of the temperature profile (see Figure 2B and 2C). Peak temperature also appears to be the predominant determinant of the phase of activity offset (see Figure 2C, and Additional file 3) which shows tight linkage to the time of peak activity. When a broad temperature peak is used, peak and offset of locomotor activity appear to synchronize to its midpoint (see Figure 2C, and Additional file 3).

In addition, other features of the activity profile do change as a result of a prolonged thermophase. In particular, activity profiles tend to assume a bimodal character with distinct morning and evening peaks. In this context, the evening activity peak shows the same phase relationship to the temperature maximum as is observed for the peak of unimodal activity profiles, while the morning activity peak occurs shortly after the temperature trough, well in advance of peak temperature. One possible interpretation of these observations is that both the phase of temperature ascent and temperature descent regulate the timing of evening activity. To further explore this possibility a 48-h temperature cycle was used that results from modifying a daily temperature gradient by extending the duration of the temperature peak by 24 h (see Figure 2D). As a result, the temperature protocol alternates entrainment days with defined phases for temperature trough, ascent and descent with days of free run at constant peak temperature. If, in fact, the mid-point of the temperature peak acted directly as the main phase determinant for entrainment, this 48-h protocol would contain conflicting signals because its mid-point of peak temperature coincides with the same time of day as its (subjective) temperature trough. However, flies showed efficient entrainment to this protocol with daily peak activity consistently occurring between the phases of (subjective) temperature ascent and descent, suggesting that the combination of these two signals acts as the phase determinant. Given the observation, noted above, that the phase of entrained locomotor activity is relatively insensitive to the shape of the daily temperature profile, the relative timing of temperature ascent and descent can apparently be detected efficiently across the different temperature protocols. Taken together, these results indicate that the phase of peak unimodal or evening activity is synchronized to coincide with the daily temperature peak by the combined action of temperature increases and decreases.

### Daily temperature gradients have a strong clock-independent effect on daily locomotor activity

**Figure 3:**
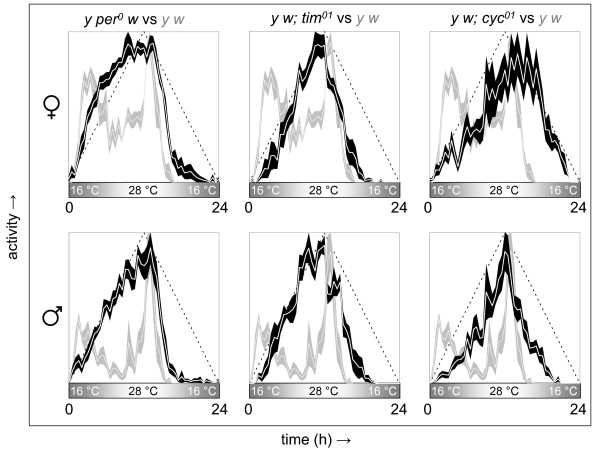
**Analysis of arrhythmic mutants uncovers strong clock-independent temperature-driven regulation of daily activity**. The daily activity profiles of flies lacking circadian clock function are readily driven to synchrony in response to daily temperature gradients. Relative to the behavioral profiles of temperature-entrained wild-type controls the mutant data show reduced bimodality (*per*^0^, *tim*^01^, and *cyc*^01^) and a more gradual decrease in activity with decreasing temperature (*cyc*^01^). Daily locomotor activity profiles (mean ± S.E.M.) in the presence of a daily temperature gradient were determined from the median activity records for groups of male and female flies carrying arrhythmic *per*^0^, *tim*^01^, or *cyc*^01 ^mutations in a *y w *genetic background (white lines with black shading) and directly compared with that of *y w *controls (white lines with gray shading). The dotted lines in the activity profiles as well as the shaded bars underneath represent the daily temperature gradient (see Z7 profiles in Additional file 2).

Although temperature affects daily behavior via synchronization of the circadian clock, as discussed above, it also modulates behavior via clock-independent mechanisms. The potential involvement of clock-independent temperature responses in shaping the daily locomotor behavior profiles observed in association with daily temperature gradients was studied in flies carrying arrhythmic clock gene alleles. The daily locomotor activity profiles of null mutants for *period *(*per*^01^) [12], *timeless *(*tim*^01^) [13], and *cycle *(*cyc*^01^) [14] were determined in the presence of a daily temperature gradient and compared with wild-type controls in a similar genetic background (*y w*). The behavioral profiles observed for arrhythmic mutant flies (see Figure 3) suggest that, in response to a daily temperature gradient, a functional circadian clock is required for anticipation of daily temperature changes, but not for the generation of diurnal activity rhythms *per se*. The temperature-driven behavior of *per*^01 ^and *tim*^01 ^mutants resembled the temperature-entrained behavior of wild-type controls in terms of the phase of peak and offset of activity, whereas the temperature-driven locomotor activity of *cyc*^01 ^mutant females showed a similar peak, but somewhat delayed activity offset. The daily activity profile of *cyc*^01 ^mutants appeared to track the phase of descending temperature more closely with approximately half of the total activity occurring during the phase of temperature descent in both males and females, suggesting that this aspect of temperature-dependent behavior may be modulated by CYC function in *per*^01 ^and *tim*^01 ^flies even in the absence of a self-sustaining oscillator. As expected, the absence of clock function in *per*^01^, *tim*^01^, and *cyc*^01 ^mutants was evident in their lack of rhythmic behavior under constant conditions and their immediate resynchronization to phase-shifted temperature gradients (data not shown). In addition, the arrhythmic mutants showed a unimodal daily pattern of activity, whereas the wild-type control flies exhibited a bimodal profile that included a morning activity peak and a subsequent period of rest in advance of the evening activity peak associated with the temperature maximum. Thus, a functional circadian clock appears to be required to generate a distinctly bimodal daily activity profile in the context of a simple daily temperature gradient. This observation is in agreement with the previously documented clock-dependent modulation of midday rest or 'siesta', which is a feature of wild-type behavior that is influenced by gender, genetic background, and the environmental protocol of temperature and light [15-17]. In this context, it is worth noting that Canton-S flies in the presence of the same temperature gradient did not reproduce the bimodal activity profile of *y w *flies (see Figure 3; data not shown), while they did exhibit bimodal temperature-entrained activity in response to a different temperature gradient (cf. Figure 2C).

### The phase of temperature-entrained circadian behavior is determined by the synchronizing daily temperature gradient as well as the relative temperature of release into free run

**Figure 4:**
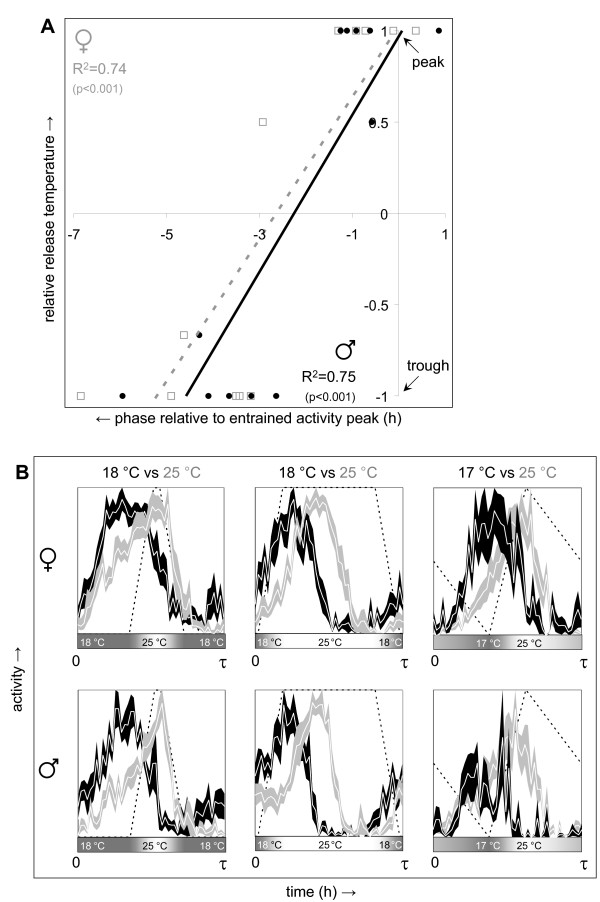
**The phase of temperature-entrained circadian behavior is modulated by the relative temperature of release into free run**. Locomotor activity was recorded for 28 groups of Canton-S flies (14 of each gender) subjected to 1 of 12 different combinations of daily temperature gradients and free running temperatures in constant darkness. The phase of circadian peak activity during free run was determined from median activity records and phase shifts were determined relative to daily peak activity during previous temperature entrainment. Phase shifts are graphed in panel **(A) **as a function of the relative temperature of release (expressed as a position in the entraining temperature range (-1 to +1)). The data is plotted separately for each gender along with trend lines and associated correlation coefficients and their significance (2-tailed). Female and male data are indicated by gray open squares with dashed lines and black filled circles with solid lines, respectively. The circadian activity profiles (mean ± S.E.M. across the circadian period length τ) in panel **(B) **illustrate phase shifts resulting from differences in relative release temperature. Profiles associated with release at the thermophase (25°C in all three examples) are indicated by white lines with gray shading, while profiles associated with cryophase release (17°C or 18°C) are indicated by white lines with black shading. The dotted lines in the activity profiles as well as the shaded bars underneath represent the daily temperature gradient used during previous entrainment. The activity profiles recorded during previous entrainment for these examples are found in Figure 2C.

Given the strong clock-independent regulation of daily locomotor activity by temperature gradients it was important to determine whether the phase relationship between peak temperature and peak activity was maintained under constant conditions. *Drosophila *maintains molecular and behavioral circadian rhythmicity over a wide range of temperatures including those used in the entrainment protocols in this study. Circadian activity profiles were determined following entrainment to one of the eight daily temperature profiles described above (Z1-8 in Additional file 2 and Additional file 3). A total of fourteen different protocols resulted from combination of these eight temperature gradients with subsequent release at different times and temperatures (see Additional files 2 and 3). As predicted based on diurnal temperature-synchronized behavior the timing of peak temperature during entrainment was found to be a key determinant of the phase of peak and offset of circadian activity. However, about half of the temperature protocols showed additional modulation of the phase of circadian activity resulting in an advance relative to the time of the subjective temperature peak by 3 to 7 h. This effect was found to be largely determined by the relationship between the free running temperature and the temperature range of prior entrainment and is summarized in Figure 4A and illustrated by some examples in Figure 4B. When flies are released from a daily temperature gradient into free run at peak temperature their activity peak and offset are maintained at essentially the same phase as during entrainment. This principle was not obviously affected by variations in amplitude, peak phase, absolute temperature, and relative duration of thermophase, cryophase, temperature ascent or descent among the different entraining temperature gradients. In contrast, release from entrainment at the temperature trough consistently resulted in a phase advance of the peak and offset of circadian activity. The behavioral phases of flies released at peak versus trough temperatures showed non-overlapping distributions with a highly significant difference (t-test *P *< 10^-6^). Additional data from flies released at intermediate temperatures is consistent with a roughly linear relationship between the relative release temperature and the resulting phase advance.

### Under 'natural' circumstances, entrainment to daily temperature gradients is predicted to reinforce light entrainment

**Figure 5:**
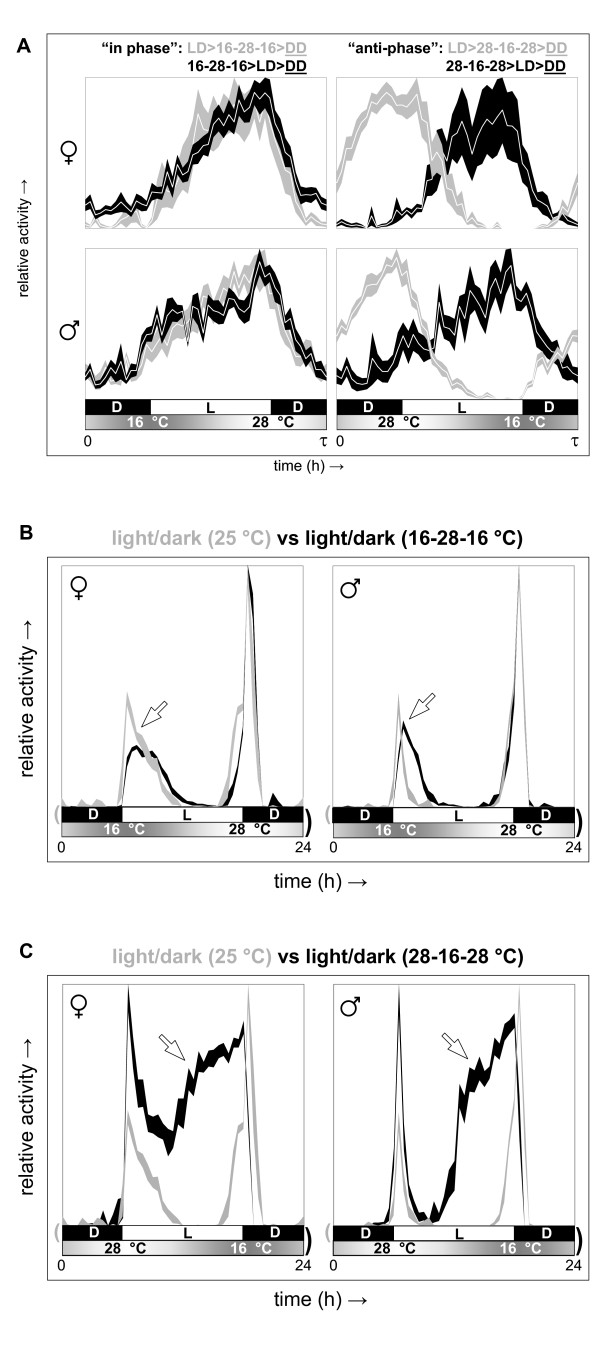
**Under 'natural' circumstances, entrainment to daily temperature gradients is predicted to reinforce light-entrainment**. **(A) **Groups of Canton-S male and female flies were sequentially subjected to 12-h light/12-h dark cycles (at constant 25°C for ≥7 days) and daily 16°C to 28°C temperature gradients (in constant darkness for ≥7 days; see profile Z7T4C4 in Additional file 2) and then released into free running conditions at 25°C in constant darkness to detect the represented circadian activity profiles. For 'in phase' alignment of the light and temperature cycles the subsequent circadian activity phase was essentially unaffected by the order of the previous treatments. In the context of 'anti-phase' alignment, however, the circadian activity phase was determined by the latter of the two environmental protocols and reversal of treatment order resulted in a shift to the opposite phase. **(B) **Simultaneous application of 'in phase' light and temperature treatments (black profiles) resulted in synchronized activity profiles similar to those found during light treatment (gray profiles) but with a more gradual increase of morning activity. **(C) **In contrast, when the same light-entrained profiles (indicated in gray) are compared with daily activity profiles representing simultaneous 'anti-phase' light and temperature treatment (indicated in black) the latter show a broad evening activity peak with a 5- to 6-h phase advance in onset as well as a relatively higher peak in morning activity. The activity profiles in all panelsrepresent the mean ± S.E.M. as measured from median activity records.

In nature, flies encounter daily temperature gradients in the presence of a light/dark cycle. Based on the natural occurrence of rising temperatures during the day and falling temperatures during the night, light and temperature entrainment were predicted to be roughly in phase if light onset and offset occurred at the same time of day as trough and peak temperature, respectively. Indeed, the circadian phase resulting from light entrainment was essentially unaltered after subsequent exposure to an in-phase daily temperature gradient and vice versa (see Figure 5A, left-hand diagrams). In contrast, when flies were sequentially exposed to oppositely aligned daily light/dark cycles and temperature gradients, circadian activity was shifted to the opposite phase during and following the second entrainment condition (see Figure 5A, right-hand diagrams). Exposure to simultaneous in-phase daily light/dark and temperature entrainment resulted in a diurnal behavior profile identical to that observed for light/dark entrainment with respect to evening activity, whereas morning activity rose more gradually upon superposition of the temperature gradient (see Figure 5B). The acute increase in morning activity observed in response to the sudden onset of bright light at constant temperature has been dubbed 'startle response' and does not depend on a functional clock [18,19]. Modulation of this aspect of morning activity by a daily temperature gradient is, therefore, not necessarily associated with a change in clock-dependent regulation. The circadian locomotor activity profile resulting after simultaneous in phase light and temperature entrainment was essentially the same as that separately observed after either type of entrainment (data not shown). Introduction of an oppositely aligned daily temperature gradient in the presence of a light/dark cycle resulted in a dramatic advancement (~5 to 6 h) of the onset of evening activity (see Figure 5C). This effect apparently required interaction between the light- and temperature-dependent entrainment mechanisms, because it produced an increase in activity at a time of day when neither light nor temperature elicited this effect on their own (see also wild-type data in Figure 3; data not shown). Moreover, when arrhythmic mutant flies were exposed to the same combined light and temperature protocol they did not show a consistent increase in activity in anticipation of the lights-off transition (see Additional file 4). In summary, these results indicate that the natural alignment of light and temperature is expected to produce cooperative entrainment of daily locomotor activity.

### Synchronization of circadian behavior to daily temperature gradients proceeds in a cumulative and relatively slow manner

**Figure 6:**
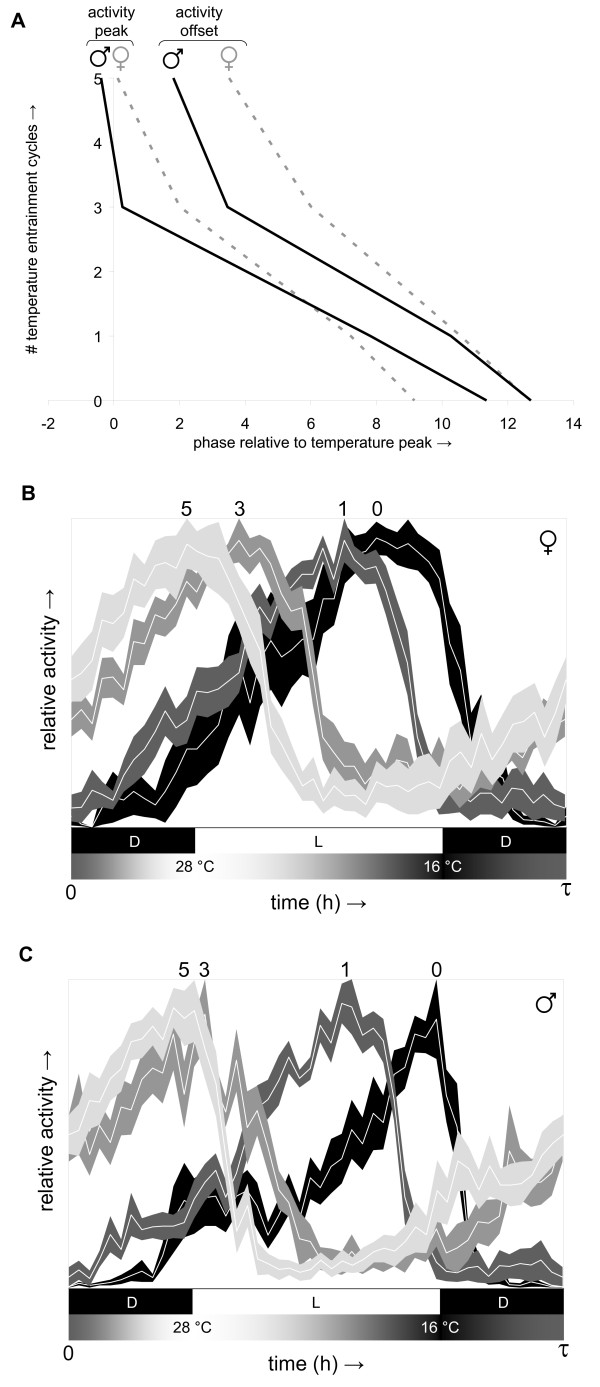
**Phase resetting responses to daily temperature gradients are relatively slow and cumulative**. After entrainment to 12-h dark/12-h light cycles (lights on from noon to midnight at constant 25°C for ≥4 days) groups of Canton-S male and female flies were exposed to 0, 1, 3, or 5 'anti-phase' daily temperature gradients (16°C at midnight to 28°C at noon to 16°C at midnight in constant darkness; see profile Z7T4C4 in Additional file 2) before being released into free running conditions at 25°C in constant darkness. The phase of peak and offset of activity during the free running circadian period length (*τ*) was determined from median activity records and plotted relative to the number of temperature cycles included in the experiment **(A)**. The corresponding circadian activity profiles (mean ± S.E.M.) for female and male Canton-S flies are represented in panels **(B) **and **(C)**, respectively, with individual experiments identified by the number of included temperature cycles (immediately above the peak) and increasingly lighter shades of gray. The shaded and black-and-white bars underneath the activity profiles represent the daily temperature gradient and light (L)/dark (D) treatments, respectively, used during previous entrainment.

In comparison with light/dark cycles daily temperature gradients provide weaker entrainment signals. While a single light/dark cycle is often sufficient to cause large phase shifts and near-complete entrainment [19,20] a single daily temperature gradient has more limited phase-resetting potential. This principle is illustrated by the experiments represented in Figure 6A, B and 6C. Light-entrained flies were exposed to zero, one, three, or five anti-phase daily temperature gradients and then released into free run. Comparison of the resulting circadian activity profiles (Figure 6B and 6C) indicated that cumulative phase advances resulted from exposure to repeated daily temperature gradients (see Figure 6A). Phase resetting was essentially complete after five days of entrainment as indicated by the circadian activity profiles resulting from prolonged exposure to the same temperature gradient (cf. Figure 5A and Figure 6B and 6C).

### Flies selectively synchronize their circadian behavior to daily temperature gradients even in the presence of continuous temperature increases or decreases

**Figure 7:**
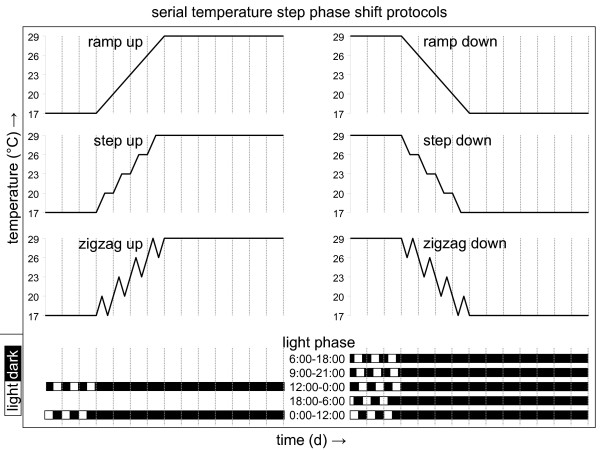
**Environmental protocols used to study temperature entrainment in the context of continuous increases or decreases in absolute temperature**. Different environmental protocols were used as indicated. First, flies were entrained to three 12-h light/12-h dark cycles at one of several phases as indicated by the white/black bars at the bottom of the panel. Next, they were subjected to a 4-day temperature regimen that superimposed a continuous temperature increase or decrease (3°C per day) on either otherwise constant temperature (ramp up or down) or daily temperature gradients with a range of 1.5°C (step up or down) or 4°C (zigzag up or down) (see Additional file 2). Note that the composite temperature profiles each show a range of 3°C on each consecutive day. Finally, the flies were released into free run at 17°C or 29°C in constant darkness. Each temperature protocol was combined with the light/dark protocols indicated below it, with the exception of 'step down' which was combined with four of the five indicated light/dark profiles (9:00 to 21:00 light phase was not used).

**Figure 8:**
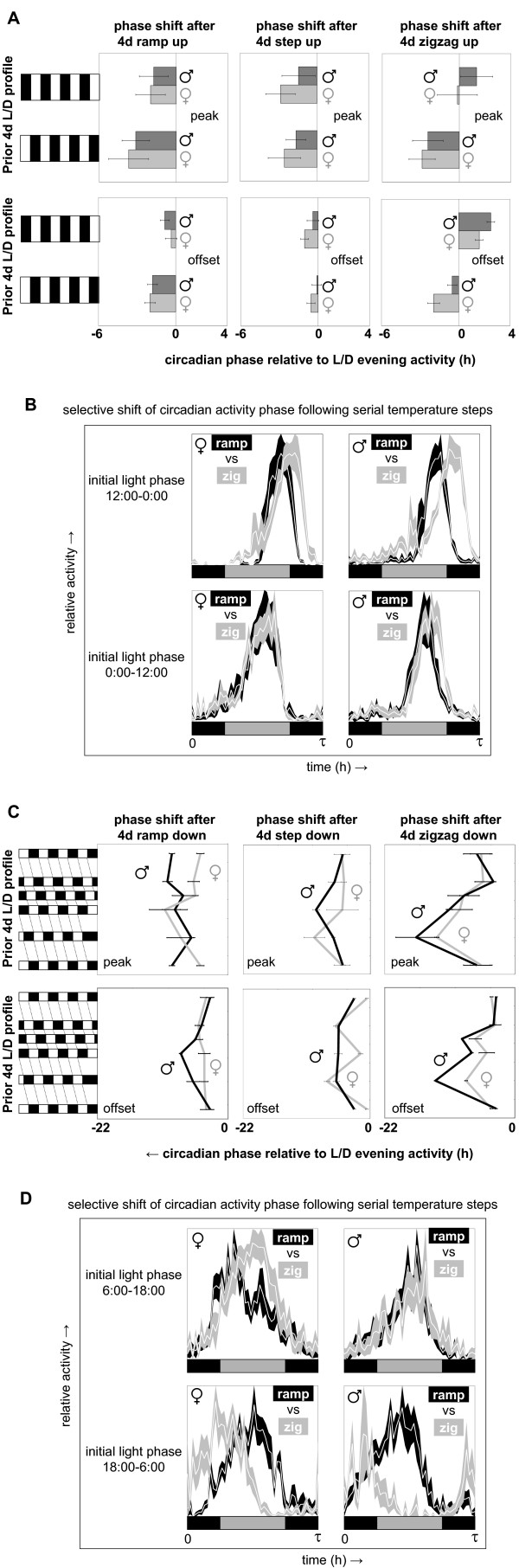
**Circadian behavior can selectively synchronize to daily temperature gradients that are combined with continuous increases or decreases in absolute temperature**. Median activity records for each experimental condition described in Figure 7 were used to generate circadian activity profiles (mean ± S.E.M.) from which the phase of peak and offset of circadian activity was then determined by interpolation. The phase shifts in peak and offset of circadian activity resulting from the various environmental protocols are summarized in panels **(A) **and **(C)**. The values and error bars indicated in these panels represent the midpoint and range of the intervals of interpolation for the phase of peak and offset. Phase-dependent resetting of the circadian activity peak and offset was only consistently observed for temperature protocols based on the 4°C daily temperature gradients ('zigzag'). This is illustrated by a side-by-side comparison of the circadian activity profiles (mean ± S.E.M.) found for the 'ramp up' and 'zigzag up' protocols after 12:00 to 0:00 and 0:00 to 12:00 light phases **(B) **and the 'ramp down' and 'zigzag down' protocols after 6:00 to 18:00 and 18:00 to 6:00 light phases **(D)**. The bars underneath the activity profiles in (B) and (D) indicate the phase of light (gray) and dark (black) during the prior light/dark cycles. Please note that, to facilitate direct comparison, the activity profiles are plotted relative to the previous light period rather than experimental time, which differs by 12 h for the top versus bottom diagrams in (B) and (D).

The temperature profiles experienced by flies in nature vary not only in a daily fashion but also due to seasonal influences and to less predictable short-term factors such as the weather and the location of the animal. Daily temperature gradients will, therefore, represent reliable and consistent synchronizing signals for daily molecular and behavioral rhythms only if they can be selectively recognized in the presence of other temperature signals. A simple test of this principle was carried out by combining daily temperature gradients with a 0°C, 1.5°C, or 4°C daily range with continuous increases or decreases (3°C per day) in absolute temperature (see Additional file 5). At the start of the experiment daily locomotor activity was entrained to selected phases with the help of light/dark cycles. Next, flies were exposed for four days to one of the six temperature protocols described above before release at constant temperature (see Figure 7). The resulting peak and offset of subsequent circadian activity were determined and compared for different phase relationships with the initial light/dark cycle. In most cases phase shifts relative to the original light-entrained evening activity peak were observed, but for the 0°C and 1.5°C daily temperature gradients these shifts were largely independent from the phase relationship with the previous light/dark cycle. Such constitutive phase changes do not result from entrainment to a daily temperature component, but rather from the effects of continuous increases or decreases in absolute temperature on activity. In contrast, phase-dependent resetting was consistently observed for protocols involving the 4°C daily temperature gradient (cf. data for zigzag versus step and ramp protocols in Figure 8A, B, C and 8D). This is nicely illustrated in a direct comparison of the circadian activity profiles resulting from exposure to simple continuous temperature increases or decreases with or without the 4°C daily temperature gradient (Figure 8B and 8D).

### Entrainment of molecular rhythms to daily temperature gradients

**Figure 9:**
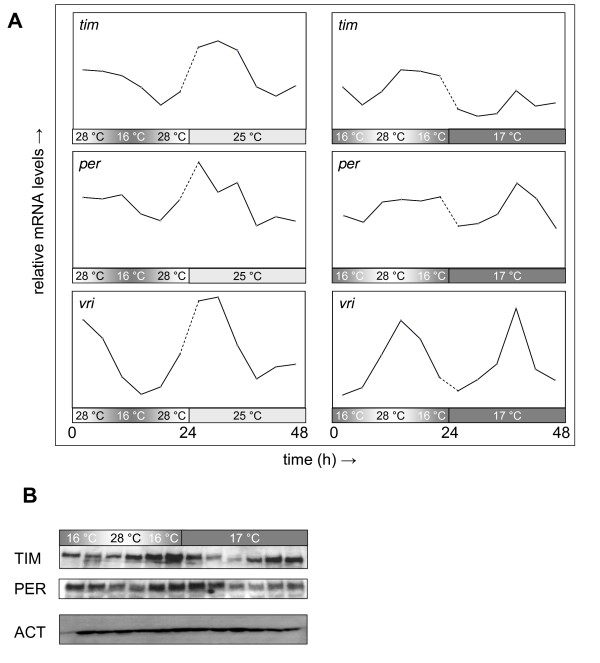
**Clock gene expression rhythms during and after entrainment to daily temperature gradients**. Canton-S flies were synchronized to a temperature gradient (16°C to 28°C) in constant darkness and then, during the experimental day, either kept in the temperature gradient or released to constant conditions at 25°C or 17°C. Fly heads were harvested 1, 5, 9, 13, 17, and 21 h after (subjective) peak temperature. Northern blot analyses for the clock-controlled transcripts *tim*, *per*, and *vri *(normalized to *rp49 *as an internal control) are represented in panel **(A)**. For comparison the entrainment data set was plotted along with each of the two free run data sets in matching phase order. Each data point was the average of one to three independent experiments. Note that the entrained molecular phases were preserved during free run at either temperature, but that *tim *transcript levels appear to be down-regulated at constant 17°C. Western blot analysis for TIM, PER and ACTIN (as an internal control) of protein extracts from heads collected in the same schedule during entrainment and free run at constant 17°C (panel **(B)**) indicated that PER and TIM protein phases maintained a normal delay (~6 h) relative to transcript levels both during entrainment to the daily temperature gradient and subsequent free run at constant 17°C. Data for a representative Western blot are shown (note that, due to a lower amount of sample, the signals for the first time point, are artificially low).

The phases of circadian gene expression resulting from entrainment to a daily temperature gradient can be predicted based on the observed alignment of molecular and behavioral circadian rhythms during and after entrainment to light/dark cycles [21]. Thus, peak expression of CLK/CYC-regulated transcripts such as *per*, *tim*, and *vri*, which coincides with the light-entrained evening locomotor activity peak at dusk, is expected to occur during the temperature-entrained evening activity peak, which falls near the temperature maximum of a synchronizing daily temperature gradient. To validate these predictions *per*, *tim*, and *vri *transcript rhythms were determined in adult head extracts during and after entrainment to a daily temperature gradient. Indeed, CLK/CYC-regulated transcript profiles show the same phase relationship to the evening locomotor activity peak after entrainment to daily temperature gradients (see Figure 9A) as previously demonstrated for light/dark cycles [20].

As part of these experiments the effect of different relative release temperatures was examined as well. During the first full day of free running conditions no obvious phase changes were observed in *per*, *tim*, or *vri *transcript rhythms regardless of the relative release temperature (see Figure 9A). However, the absolute levels of *tim *transcript were reduced upon release at relatively cold temperatures. Temperature-entrained PER and TIM protein phases maintained a normal delay (~6 h) relative to transcript levels both during exposure to the daily temperature gradient and during subsequent free run at constant 17°C (see Figure 9B).

## Conclusion

The present study systematically examined the entrainment of clock-controlled behavior to daily environmental temperature gradients. As a result, a number of key properties of circadian temperature entrainment were identified. Collectively, these properties, as described below, represent a circadian temperature entrainment mechanism that is optimized in its ability to detect the time-of-day information encoded in natural environmental temperature profiles.

First, daily temperature gradients show a remarkable efficiency at entraining behavioral and molecular daily rhythms in *Drosophila*. The minimal range identified here for efficient entrainment by temperature gradients (1.5 to 4°C) is similar to that previously reported for square wave temperature cycles (2 to 3°C; [19,22]). Temperature input pathways, therefore, appear to be more than sensitive enough to respond to a wide variety of daily environmental temperature profiles.

Second, synchronization of circadian locomotor behavior is associated with detection of changes in relative temperature rather than absolute temperature. Circadian phase showed comparatively little dependence on absolute temperature, but was, instead, reliably predicted based on the phase of the relative temperature peak and the relative temperature of release. The use of a diverse panel of daily temperature wave forms and associated release temperatures in this study allowed this property of circadian temperature entrainment to be recognized. A crucial benefit of entrainment to relative temperature changes is that circadian clocks can effectively respond to daily temperature oscillations in the presence of seasonal (or other) changes in average temperature.

Third, the daily temperature peak as defined by the alignment of both ascending and descending daily temperature phases sets the circadian phase of entrainment. Peak circadian locomotor activity was strongly linked to the configuration of the ascending and descending temperature phases during entrainment. And this association was maintained irrespective of the phase of other features of the synchronizing daily temperature profile such as the trough. Again, analysis of a diverse panel of daily temperature wave forms in the present study was instrumental in allowing this property to be identified. A combined role of temperature increases and decreases in the circadian entrainment of eclosion in *Drosophila pseudoobscura *populations was proposed in an early study by Zimmerman *et al*. [23]. It is, therefore, very well possible that circadian temperature entrainment of locomotor activity and eclosion make use of the same mechanism.

Fourth, the relative temperature of release into free run modulates circadian phase. Whereas release into free running conditions at a temperature matching the entrainment peak has no apparent effect on circadian phase, progressive phase advances result from release at temperatures approaching the entrainment trough. Thus, changes in average daily temperature modulate rather than block entrainment to daily temperature gradients. This arrangement may allow for the simultaneous integration of both seasonal and daily information in generating rhythmic behavior. Seasonal modulation of circadian behavior occurs in part as a result of temperature sensitive splicing of the last intron of the clock gene *period *[16]. Indeed, lower temperatures directly induce more efficient splicing and, as a result, an advanced molecular and behavioral phase [17,24]. Although temperature-dependent splicing of *per *may have contributed to the phase advances observed upon release at lower absolute temperatures this mechanism cannot fully account for the observed sensitivity to relative temperature changes. Behavioral phase advances were observed upon release at constant trough temperature even for mutants that block *per *splicing (see Additional file 6). Moreover, no obvious molecular phase advance in *per *transcript or protein accumulation was detected for wild-type flies released at low relative temperature (see Figure 9A and 9B, above).

Fifth, circadian phase responses to daily temperature profiles occur slowly and often require several days to be completed. Daily temperature gradients are weaker synchronizers of circadian behavior than light/dark cycles and have a more limited phase shifting potential per cycle. As a result, large temperature-dependent phase shifts are not triggered by single, anecdotal fluctuations in temperature, but rather by repeated exposure to convergent phase-shifting signals over the course of a number of days. The *Drosophila *clock is, therefore, tuned to repeated daily temperature components and able to resist dramatic short-term responses to irregular fluctuations in environmental temperature. Given the complexity and irregularity of natural environmental temperature profiles, this appears to be a very useful adaptation. The temperature-dependent phase responses described here are comparable to those reported earlier for square-wave temperature pulses and cycles [21,25,26]. For example, Boothroyd and colleagues found that it took flies 5 days to complete a 10-h phase advance in response to a square wave 18°C/25°C temperature cycle, whereas Busza *et al*. observed that 8-h phase advances and 8-h delays in response to a 20°C/29°C square wave were achieved over a time frame of more than 6 days or 5 days, respectively [21,25].

Sixth, daily cycles in relative temperature can synchronize circadian behavior even when they are presented as part of more complex temperature profiles. Entrainment to daily temperature gradients persisted even in the context of continuous gradual temperature increases or decreases. To effectively detect the time-of-day information encoded in environmental temperature profiles circadian clocks have to be able to sense daily changes in relative temperature in the context of patterns that contain unrelated thermal signals, for example, due to weather-related developments. The present study shows that this is, indeed, the case in examples where continuous changes in absolute temperature are used as a back-drop for entrainment.

Seventh, naturally aligned daily temperature and light cycles provide cooperative circadian entrainment. Daily temperature gradient and light/dark entrainment reinforced each other if the phases of ascending and descending temperature were in their natural alignment with the light and dark phases, respectively. In nature, entrainment of the *Drosophila *circadian clock to temperature profiles occurs in the context of a daily light/dark cycle. To achieve the highest sensitivity towards environmental time-of-day information the circadian clock is expected to show light/dark-entrained and temperature-entrained phases that approximately coincide when these zeitgebers are in their natural alignment. The phase determinants of temperature and light entrainment both result from changes in solar irradiance and normally occur in succession during the second half of the day. Peak temperature, the main synchronizing signal for thermal entrainment (see above), generally falls in the afternoon several hours in advance of dusk [27], which is the major phase determinant of light entrainment (for example, [20,28]). This natural association of temperature and light-dependent phase resetting likely facilitates cooperative entrainment. As shown here, separate or combined treatments with daily light/dark cycles and temperature gradients produce similar circadian behavioral phases when the phases of ascending and descending temperature match those of light and dark, respectively. In this arrangement, as in natural conditions, the daily temperature minimum coincides with the time just before dawn, while the temperature maximum is associated with a time point in the second half of the day.

What might be the molecular mechanism underlying the circadian temperature synchronization described in this study? Analysis of temperature-entrained gene expression indicates that the phase relationship between behavioral circadian rhythms and molecular rhythms in the fly head is relatively stable and similar to that observed previously for light entrainment. Peak expression of CLK/CYC regulated transcripts (*per, tim, vri*) occurs shortly after peak temperature and PER and TIM protein expression show the expected ~6-h delay relative to their transcript levels [29]. These observations confirm and complement analyses from a previous study comparing the genome-wide transcript profiles in the adult head during and after treatment of wild-type and arrhythmic mutant flies with daily light/dark or square-wave temperature cycles [21]. In that study many circadian transcript profiles were identified that showed the same fixed relationship between light-entrained and temperature-entrained circadian phases, indicating that the same molecular clock mechanism is responsible for both light-entrained and temperature-entrained gene expression rhythms. Moreover, the expression levels of clock gene transcripts (*per, tim, Clk, vri, Pdp1-ε, cry*) and proteins (PER, TIM) were found to respond to environmental temperature changes in a clock-dependent manner [21,30]. The identification here of the peak temperature phase as the main determinant of the temperature-entrained behavioral phase suggests that associated molecular events are responsible for temperature entrainment. Perhaps the two most relevant of these are the peak in transcript levels for CLK/CYC-regulated transcripts, including *per *and *tim*, and the accelerated accumulation of PER and TIM proteins. Temperature-dependent modulation of circadian phase has been related to changes in *per *transcript levels as well as PER and TIM protein levels [16,21,24,26,30-34].

As mentioned before, *per *transcript levels are known to be regulated by temperature-dependent splicing of the terminal intron of *per *and this, in turn, affects PER protein levels as well as locomotor activity in the evening [16,24]. At lower constant temperatures splicing of this intron is enhanced, which leads to earlier accumulation of *per *mRNA and protein and advanced evening activity. This mechanism does, however, not appear to be required for synchronization of the clock to daily temperature cycles. Entrainment of circadian behavior to daily temperature cycles presented in constant darkness was largely unaffected in mutants that prevent splicing of the terminal *per *intron (Additional file 6). In addition, *per *splicing levels in wild-type flies did not show obvious daily changes in temperature cycles during constant light [26].

Complex formation of PER and TIM protein with CRYPTOCHROME (CRY) [34] and destabilization of TIM and PER protein [32] have been implicated in circadian phase resetting in response to heat shock treatment. However, these responses can be separated from entrainment to daily temperature cycles based on the requirement of the former for treatment with high temperatures (>34°C) and the involvement of CRY [32,34] which are both dispensable for synchronization to daily temperature cycles [19,21,25,26,33,34]. Moreover, heat shock treatment preferentially results in phase delays, whereas both phase advances and delays are readily observed in response to daily temperature cycles ([25,34]; data not shown).

Changes in TIM protein levels in a subset of clock neurons (type 2 Dorsal Neurons, DN2s, and Lateral Posterior Neurons, LPNs, and, to a lesser extent, type 1 and 3 Dorsal Neurons, DN1s and DN3s) were reported in association with a shifted alignment of simultaneously applied light/dark and temperature cycles [31]. It is not yet clear, however, whether these changes could be responsible for the advanced onset of evening activity recorded when the thermophase and cryophase of a square-wave temperature cycle were centered around the lights-on and lights-off transitions, respectively [31]. Observations resulting from the combination of a light/dark cycle with an anti-phase daily temperature gradient described here, for wild-type, but not arrhythmic mutant flies, indicate that the advance in evening activity onset is a clock-dependent response rather than a startle response following a sudden temperature decrease. Apart from a possible temperature-specific role for the LPNs, the set of clock neurons responsible for circadian temperature entrainment mostly coincides with the set known to control circadian light entrainment [25]. In this context, the subtle changes in TIM expression in the DN1s, which have been reported to modulate evening activity in the context of light treatment [35-37], may merit further examination.

Thus, although there is precedent for the temperature-dependent regulation of circadian phase via modulation of either *per *transcript levels or PER and TIM protein levels, neither of these mechanisms has been conclusively shown to account for circadian synchronization to environmental temperature cycles. Further molecular studies tracking the dynamics of circadian transcript and protein expression as well as post-translational modifications during temperature cycle-dependent phase advances and delays are expected to provide insight into this issue.

In contrast to *Drosophila *and other poikilothermic organisms, homeothermic mammals such as humans do not have a body temperature that directly tracks environmental temperature. Nevertheless, there is evidence supporting the involvement of daily temperature cycles in the maintenance of mammalian circadian rhythms because body temperature itself not only exhibits daily clock-controlled rhythms but can also act as a synchronizing cue [38,39]. Two pathways in particular have been proposed to be involved in mediating entrainment to mammalian body temperature cycles: (1) Heat Shock Factor 1 (HSF1)-dependent transcriptional regulation and (2) control of gene expression by cold-induced RNA binding proteins [39]. In this context, it is intriguing that rhythmic expression of HSF1-regulated heat shock proteins genes was not only observed in the mouse liver [40], but also in the heads of *Drosophila *exposed to a synchronizing daily temperature cycle of modest amplitude [21]. Additional research is needed to address the potentially conserved role of these genes in temperature entrainment.

## Methods

### Drosophila strains

The (behaviorally) wild-type *Drosophila melanogaster *strains used were Canton-S, and *yellow white *(*y w*); in addition, comparative analyses were performed for *y per*^01^*w, y w; tim*^01^, and *y w;; cyc*^01 ^arrhythmic mutant flies. The flies were raised on standard yeast cornmeal agar medium.

### Behavioral analysis

Individual flies were monitored in glass tubes on standard sugar agar media (5% sugar, 1% agar plus 0.07% Tegosept [Genesee Scientific, San Diego, CA] to inhibit fungal growth) and their locomotor activity was analyzed with the Drosophila Activity Monitoring System (DAMSystem; TriKinetics, Waltham, MA) housed in Percival I-36VL incubators, Percival Scientific, Perry, IA) equipped with fluorescent lights, humidity control, and Advanced Intellus Control systems to generate ramping temperature profiles. Unless otherwise specified, temperature entrainment and free running analyses were conducted in constant darkness at 70% relative humidity. Individual and median activity records, as well as periodic activity profiles, time series, and periodograms were generated using ClockLab Software (ActiMetrics, Wilmette, IL). Median activity records were created from the raw individual activity records on a point-by-point basis without prior normalization. Activity profiles representing the average of the median activity records (± S.E.M.) across the included intervals of 24 h or the intrinsic circadian period length *τ *were generated from flies stably synchronized to an environmental protocol or under free running conditions, respectively. Data from days spanning the transition from synchronizing to free running conditions or prior to stable synchronization were excluded from the activity profile analyses. Intrinsic circadian period length *τ *was determined by conducting a chi-square periodogram analysis of median activity data using 0:00 am on the first full day of free running conditions (as detected by the DAMSystem software) as starting point. The average activity profiles during synchronizing conditions were plotted relative to the following reference points at *t *= 0: DAMSystem time 0:00 am (Figure 1, left-hand diagrams; Figure 2C, left-hand and right-hand diagrams; Figure 3; top diagram in Additional file 1), 6:00 am (Figure 5B and 5C; Additional file 2), and 9:00 pm (Figure 2C, center diagrams). The average activity profiles during free running conditions were plotted relative to 0:00 am (DAMSystem time) on the first full day of free run with the following shifts in circadian phase angle at *t *= 0: unshifted (Figure 1, right-hand diagrams; Figure 4B left-hand and right-hand diagrams; Figure 8D top diagrams; bottom diagram in Additional file 1), 90° advance (Figure 5A; Figure 6B and 6C; Figure 8B top diagrams), 45° delay (Figure 4B center diagrams), 90° delay (Figure 8B bottom diagrams), and 180° change (Figure 8D bottom diagrams). The phase of the peak and offset of activity during and following exposure to daily environmental cycles was determined from the activity profiles by interpolation as illustrated by Additional file 1. Because the activity profiles for free running data do not represent the time from the onset of free run during the day of transition and 0:00 am (DAMSystem time) on the first full day of free run, a correction had to be used to calculate free running phases relative to the previous environmental cycle. Specifically, if the flies were under free-running conditions for *n *hours on the day that the transition from entrainment to free run took place and they showed a subsequent free running period length *τ*, a phase correction of [*n *× (24 – *τ*)]/24 h of circadian time was applied. These corrections have been incorporated in Figures 4A, 6A, 8A and 8C as well as Additional files 3 and 7. Numbers of flies (*n*) and days (*d*) used to generate median activity records and activity profiles are indicated in Figure 1, and Additional files 3 and 7, or as follows.

Figure 3 (*n *× *d*): *y w *♀ (11 × 7), *y w *♂ (15 × 7), *y per*^01^*w *♀ (16 × 10), *y per*^01^*w *♂ (16 × 10), *y w; tim*^01^*w *♀ (9 × 7), *y w; tim*^01 ^♂ (5 × 7), *y w;; cyc*^01^*w *♀ (14 × 9), *y w;; cyc*^01 ^♂ (13 × 9).

Figure 5 (*n *× *d*): (A) 'in phase' 16-28-16°C > LD ♀ (6 × 10), ♂ (4 × 10), 'in phase' LD > 16-28-16°C ♀ (3 × 10), ♂ (7 × 10), 'anti-phase' 16-28-16°C > DL ♀ (6 × 10), ♂ (3 × 10), 'anti-phase' DL > 16-28-16°C ♀ (5 × 10), ♂ (6 × 10); (B) LD 25°C ♀ (6 × 7), ♂ (8 × 7), LD 16-28-16°C ♀ (8 × 10), ♂ (6 × 10), (C) LD 25°C ♀ and ♂ (same data as in (B)), LD 28-16-28°C ♀ (7 × 11), ♂ (7 × 11).

Figure 6 (*n *× *d*): (B) ♀ after 0, 1, 3, and 5 anti-phase temperature gradients: (8 × 10), (7 × 10), (7 × 10), and (8 × 10), respectively; (C) ♂ after 0, 1, 3, and 5 anti-phase temperature gradients: (6 × 10), (7 × 10), (8 × 10), and (8 × 10), respectively.

Additional file 4: LD 25°C *y per*^01^*w *♀ (16 × 8) and ♂ (13 × 8), *y w; tim*^01^*w *♀ (5 × 8) and ♂ (3 × 8), *y w;; cyc*^01^*w *♀ (12 × 8) and ♂ (15 × 8), LD 28-16-28°C *y per*^01^*w *♀ (16 × 6) and ♂ (16 × 6), *y w; tim*^01^*w *♀ (9 × 6) and ♂ (5 × 6), *y w;; cyc*^01^*w *♀ (14 × 7) and ♂ (13 × 7).

Additional file 6: *y per*^01^*w;; {perG1} *♀ DD 25°C (8 × 8), DD 17°C (7 × 9), and ♂ DD 25°C (8 × 8), DD 17°C (8 × 9), *y per*^01^*w; {perA17} *♀ DD 25°C (3 × 8), DD 17°C (5 × 8), and ♂ DD 25°C (7 × 8), DD 17°C (8 × 8), *y per*^01^*w;; {perB'2} *♀ DD 25°C (4 × 8), DD 17°C (4 × 9) and ♂ DD 25°C (4 × 8), DD 17°C (4 × 9).

### Northern blot analysis

Total RNA was extracted from approximately 100 μl of adult heads per time point using guanidinium thiocyanate followed by centrifugation in Cesium chloride solution as previously described [41]. Northern analysis was performed according to published protocols [21]. Radioactive signals on the blots were visualized and quantitated with a Storm 840 Phosphorimager (GE healthcare, Piscataway, NJ) and the results plotted in Microsoft Excel (Microsoft Corporation, Redmond, WA).

### Western blot analysis

Total protein was extracted from adult heads and subject to Western blotting analysis with anti-ACTIN (Sigma A 2066), Sigma-Aldrich (St Louis, MO) anti-PER, and anti-TIM antisera as previously described [21]. Specificity of the detected PER and TIM protein signals was verified by inclusion of an extract from *y per*^01^*w; tim*^01 ^flies on the same blot (not shown).

## Abbreviations

CLK/CYC: CLOCK/CYCLE; CRY: CRYPTOCHROME; DD: constant dark; DN1, 2, 3: type 1, 2, 3 Dorsal Neuron; LPN: Lateral Posterior Neuron.

## Authors' contributions

HW conceived of the study, designed the experiments, carried out behavioral studies and data analysis, and drafted the manuscript. JC contributed to behavioral and Northern analyses. TG conducted Western analyses. All authors read and approved the final manuscript.

## Supplementary Material

Additional file 1 **Figure S1**. Determination of peak and offset of locomotor activity. In this example the 12 female Canton-S flies from Figure 1 are used as an example. First individual activity records were compared for uniformity to ensure that the generation of median data did not obscure the presence of strong divergent trends in subsets of individuals. Next, median locomotor activity was determined. The daily activity profile representing temperature entrainment was generated by averaging median daily activity for the experimental days following stable synchronization of behavior and then plotting mean ± S.E.M. activity relative to a 24-h period. A modified procedure was used to generate the daily activity profile representing free running conditions. In this case the presence of significant circadian rhythmicity during the free running days immediately following entrainment was tested by chi-square periodogram analysis (using the CLOCKLAB software package). If significant circadian periodicity (*P *< 0.01) was detected, median daily activity was averaged for the specified circadian period length and plotted as mean ± S.E.M. activity relative to the circadian period (τ). The phases of daily activity peak and offset were determined using interpolation. The phase interval during which the (mean + S.E.M.) activity value was equal or higher than highest mean activity value observed was used to define the peak (as the mid-point of this interval). The phase interval following the peak during which the half-maximal activity value (50% of the maximal (mean + S.E.M.) value) fell within the observed mean ± S.E.M. activity range was used to define the offset (as the mid-point of this interval). In case of bimodal profiles the evening activity peak was used for this analysis. Click here for file

Additional file 2 **Figure S2**. Daily temperature gradient protocols used to identify the phase determinants of circadian temperature entrainment. The graphs represent the experimental temperature protocols used in the analyses of Figures 2 and 4. These protocols are depicted during the last day of entrainment to one of eight daily temperature gradients, a day of transition, and the first day of free running conditions at constant temperature, all carried out in constant darkness. Note that there were eight different entrainment profiles (Z1-8; represented in Figure 2A and 2B) and four different free running conditions at constant temperature (C1-4) that were used together in 12 different combinations (represented in Figure 4A). Because two pairs of protocols (Z7T1C2 and Z7T2C2; Z7T3C4 and Z7T4C4) combined the same entrainment and free running conditions, but differed in the daily timing of the transition into free run the total number of different protocols was 14. Temperature ranges during entrainment: 14 to 22.5°C (Z1), 17 to 25°C (Z2), 18 to 25°C (Z3, Z4, Z5, Z6), 16 to 28°C (Z7), and 22 to 30°C (Z8). Maximal rate of change during entrainment: 1.4°C/h (Z1), 1.3°C/h (Z2), 1.75°C/h (Z3, Z6), 0.6°C/h (Z4), 0.8°C/h (Z5), and 1°C/h (Z7, Z8). Free running temperatures: 17°C (C1), 18°C (C2), 22°C (C3), 25°C (C4). For profiles Z3C2, Z6C4, and Z4C2, the conditions during the transition days were identical to those of free running conditions. Additional file 3 contains the incubator programs used to generate each protocol as well as behavioral data representing each protocol or combination of protocols. Click here for file

Additional file 3 **Table TS1**. This table contains the experimental data represented in Figures 2A and 2B and 4A (as indicated by color coding) as well as detailed information for each experimental condition regarding the temperature protocols, number and gender of flies, free running period length (tau), and estimated peak and offset phases during entrainment and free running conditions. The indicated corrected free running phases reflect adjustments for free running intervals occurring on the day of transition that were not accounted for in the activity profiles (see *Methods*). Click here for file

Additional file 4 **Figure S3**. Daily temperature gradients produce strong clock-independent behavioral responses even in the presence of light/dark cycles. The six panels with daily activity profiles in this figure represent the mean ± S.E.M. during 12-h light/12-h dark conditions at constant 25°C (gray shading) or 12-h light/12-h dark conditions in the presence of a daily 28°C to 16°C to 28°C temperature gradient (black shading) as determined from median activity records of *y per*^0^*w*, *y w; tim*^01^, and *y w;; cyc*^01 ^flies. In the presence of a light/dark cycle at constant temperature *per*^01 ^and *tim*^01 ^flies showed startle responses to both light/dark transitions, while *cyc*^01 ^flies exhibited a reduced startle response that was detected only at the lights-off transition. In addition, *cyc*^01 ^males showed paradoxical masking (that is, increased activity during the dark) under these conditions. Consistent with their reduced light sensitivity and robust temperature-driven responses, *cyc*^01 ^flies showed activity profiles that tracked daily temperature gradients even in the presence of light/dark cycles. The activity profiles of *per*^01 ^and *tim*^01 ^flies in the presence of the combined light and temperature protocol were similar, but contained a number of additional light-dependent features (cf. black traces with *per*^01 ^and *tim*^01 ^profiles in Figure 3): First, the *per*^01 ^and *tim*^01 ^activity profiles under these conditions showed a more gradual decrease in activity in the presence of the anti-phase light/dark cycle than in constant darkness, second, *per*^01 ^and *tim*^01 ^males showed an acute and temporary light-dependent down-regulation of activity after the lights-on transition (as indicated by '*'), and, third, the *tim*^01 ^flies showed suppressed activity for 5 to 6 h following the lights-off transition (as indicated by '#'). Click here for file

Additional file 5 **Figure S4**. Composite temperature profiles combining both daily temperature gradients and continuous increases or decreases. The left and right panels illustrate how the composite temperature profiles used for the experiments in Figure 7 resulted from combinations of daily temperature gradients with gradual 3°C temperature increases or decreases, respectively. The profiles in the first row represent combinations with constant temperature, whereas the profiles in the second and third rows involved daily temperature gradients with a 1.5°C or 4°C range, respectively. Click here for file

Additional file 6 **Figure S5**. The phase of temperature-entrained circadian behavior is modulated by the relative temperature of release into free run in the absence of thermosensitive splicing of the terminal intron of the *per *gene. The introduction of mutations that block temperature-dependent splicing of *per *did not prevent temperature-dependent modulation of the circadian phase of locomotor activity in male flies. Female circadian behavior appeared to be largely insensitive to the relative temperature during free running conditions, but this appeared to be due to the genotype used for transgenic rescue of the *per*^01 ^mutation and unrelated to thermosensitive splicing of *per*. Temperature-dependent circadian locomotor behavior was determined for flies in which the *per*^01 ^mutation was rescued by transgenes containing either wild-type genomic sequence (*perG*) or modified alleles that lack (*perB'*) or constitutively maintain the terminal intron (*perA*), such that splicing at this intron was blocked. Following entrainment to a daily temperature gradient (ranging between 17°C and 25°C in constant dark (DD) conditions) flies were released under free running conditions at constant peak (25°C) or trough temperature (17°C) conditions. The diagrams represent median locomotor activity profiles relative to the intrinsic circadian period, tau. The dotted lines inside as well as the shading in the horizontal bars below the activity profiles represent the previous daily temperature gradient. Click here for file

Additional file 7 **Table TS2**. This table contains the experimental data represented in Figure 8A and 8C, as well as detailed information for each experimental condition regarding the light and temperature protocols, number and gender of flies, free running period length (tau), and estimated peak and offset phases during entrainment and free running conditions. The indicated corrected free running phases reflect adjustments for free running intervals occurring on the day of transition that were not accounted for in the activity profiles (see *Methods*). Only the 'step up' and 'step down' protocols included an interval (12 h) of unaccounted free run on the transition day. No correction was required for the 'ramp' and 'zigzag' protocols. Click here for file
