# Supplementary figures and images for: Selective entrainment of the Drosophila circadian clock to daily gradients in environmental temperature
Source: BMC Biol. 2009 Aug 11;7:49. doi: 10.1186/1741-7007-7-49 (PMC2745372; doi:10.1186/1741-7007-7-49)

**S1**

individual  
activity records

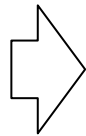

median  
activity record

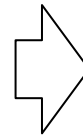

mean daily  
activity profile

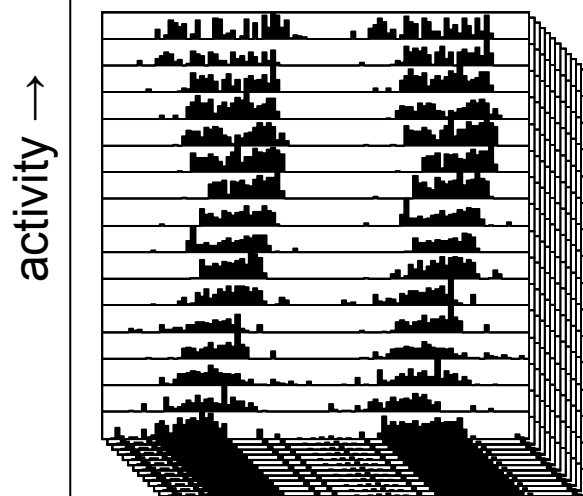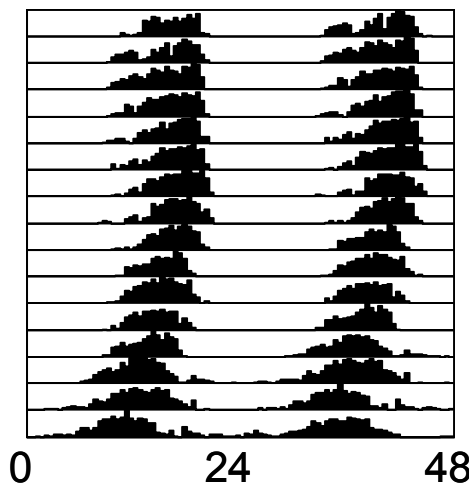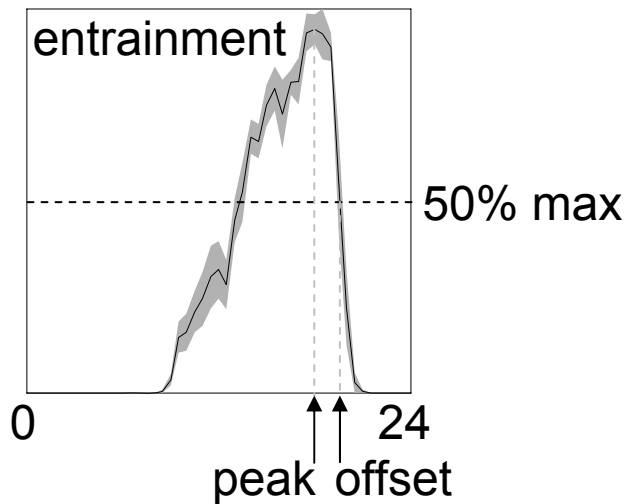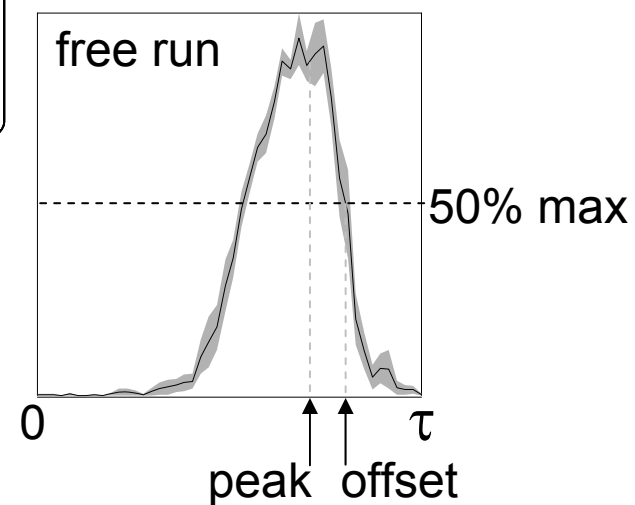

time (h) →

Supplement: Additional file 1 — Figure S1. Determination of peak and offset of locomotor activity. In this example the 12 female Canton-S flies from Figure 1 are used as an example. First individual activity records were compared for uniformity to ensure that the generation of median data did not obscure the presence of strong divergent trends in subsets of individuals. Next, median locomotor activity was determined. The daily activity profile representing temperature entrainment was generated by averaging median daily activity for the experimental days following stable synchronization of behavior and then plotting mean ± S.E.M. activity relative to a 24-h period. A modified procedure was used to generate the daily activity profile representing free running conditions. In this case the presence of significant circadian rhythmicity during the free running days immediately following entrainment was tested by chi-square periodogram analysis (using the CLOCKLAB software package). If significant circadian periodicity (P < 0.01) was detected, median daily activity was averaged for the specified circadian period length and plotted as mean ± S.E.M. activity relative to the circadian period (τ). The phases of daily activity peak and offset were determined using interpolation. The phase interval during which the (mean + S.E.M.) activity value was equal or higher than highest mean activity value observed was used to define the peak (as the mid-point of this interval). The phase interval following the peak during which the half-maximal activity value (50% of the maximal (mean + S.E.M.) value) fell within the observed mean ± S.E.M. activity range was used to define the offset (as the mid-point of this interval). In case of bimodal profiles the evening activity peak was used for this analysis. [file 1741-7007-7-49-S1.pdf]

**S2**

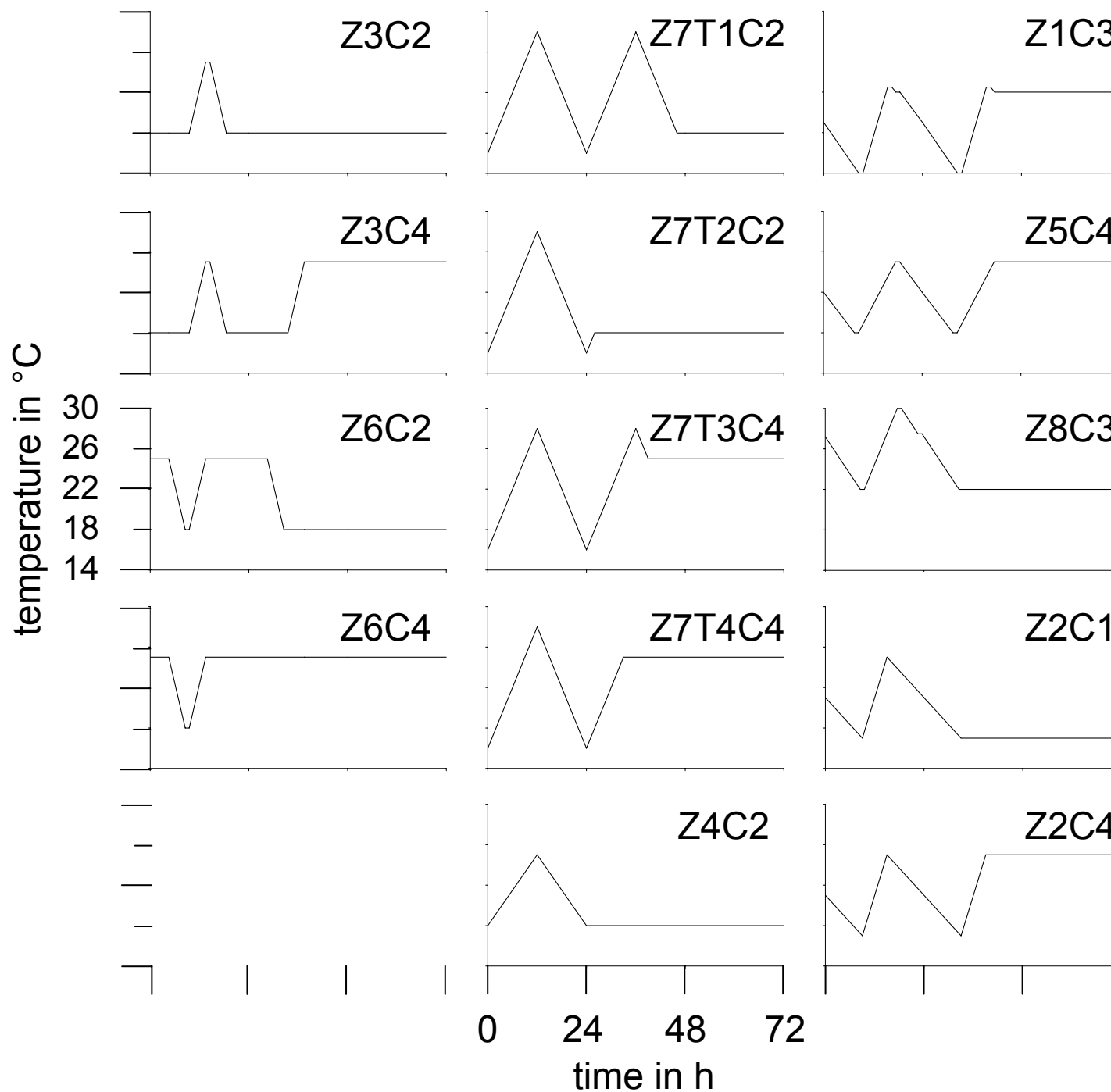

Supplement: Additional file 2 — Figure S2. Daily temperature gradient protocols used to identify the phase determinants of circadian temperature entrainment. The graphs represent the experimental temperature protocols used in the analyses of Figures 2 and 4. These protocols are depicted during the last day of entrainment to one of eight daily temperature gradients, a day of transition, and the first day of free running conditions at constant temperature, all carried out in constant darkness. Note that there were eight different entrainment profiles (Z1-8; represented in Figure 2A and 2B) and four different free running conditions at constant temperature (C1-4) that were used together in 12 different combinations (represented in Figure 4A). Because two pairs of protocols (Z7T1C2 and Z7T2C2; Z7T3C4 and Z7T4C4) combined the same entrainment and free running conditions, but differed in the daily timing of the transition into free run the total number of different protocols was 14. Temperature ranges during entrainment: 14 to 22.5°C (Z1), 17 to 25°C (Z2), 18 to 25°C (Z3, Z4, Z5, Z6), 16 to 28°C (Z7), and 22 to 30°C (Z8). Maximal rate of change during entrainment: 1.4°C/h (Z1), 1.3°C/h (Z2), 1.75°C/h (Z3, Z6), 0.6°C/h (Z4), 0.8°C/h (Z5), and 1°C/h (Z7, Z8). Free running temperatures: 17°C (C1), 18°C (C2), 22°C (C3), 25°C (C4). For profiles Z3C2, Z6C4, and Z4C2, the conditions during the transition days were identical to those of free running conditions. Additional file 3 contains the incubator programs used to generate each protocol as well as behavioral data representing each protocol or combination of protocols. [file 1741-7007-7-49-S2.pdf]

activity in light/dark (25 °C) vs light/dark (28-16-28 °C) ↑

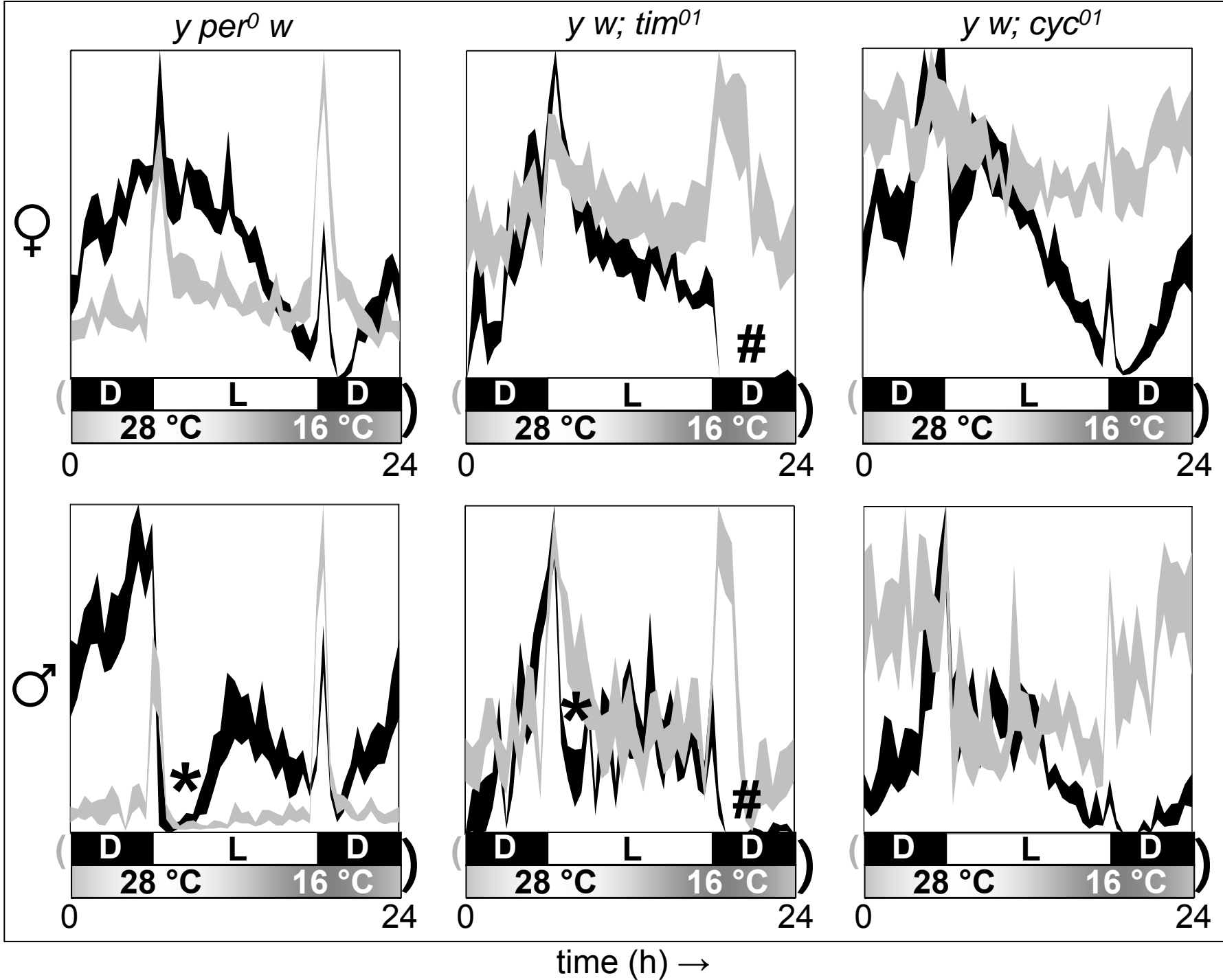

Supplement: Additional file 4 — Figure S3. Daily temperature gradients produce strong clock-independent behavioral responses even in the presence of light/dark cycles. The six panels with daily activity profiles in this figure represent the mean ± S.E.M. during 12-h light/12-h dark conditions at constant 25°C (gray shading) or 12-h light/12-h dark conditions in the presence of a daily 28°C to 16°C to 28°C temperature gradient (black shading) as determined from median activity records of y per0w, y w; tim01, and y w;; cyc01 flies. In the presence of a light/dark cycle at constant temperature per01 and tim01 flies showed startle responses to both light/dark transitions, while cyc01 flies exhibited a reduced startle response that was detected only at the lights-off transition. In addition, cyc01 males showed paradoxical masking (that is, increased activity during the dark) under these conditions. Consistent with their reduced light sensitivity and robust temperature-driven responses, cyc01 flies showed activity profiles that tracked daily temperature gradients even in the presence of light/dark cycles. The activity profiles of per01 and tim01 flies in the presence of the combined light and temperature protocol were similar, but contained a number of additional light-dependent features (cf. black traces with per01 and tim01 profiles in Figure 3): First, the per01 and tim01 activity profiles under these conditions showed a more gradual decrease in activity in the presence of the anti-phase light/dark cycle than in constant darkness, second, per01 and tim01 males showed an acute and temporary light-dependent down-regulation of activity after the lights-on transition (as indicated by '*'), and, third, the tim01 flies showed suppressed activity for 5 to 6 h following the lights-off transition (as indicated by '#'). [file 1741-7007-7-49-S4.pdf]

# S4

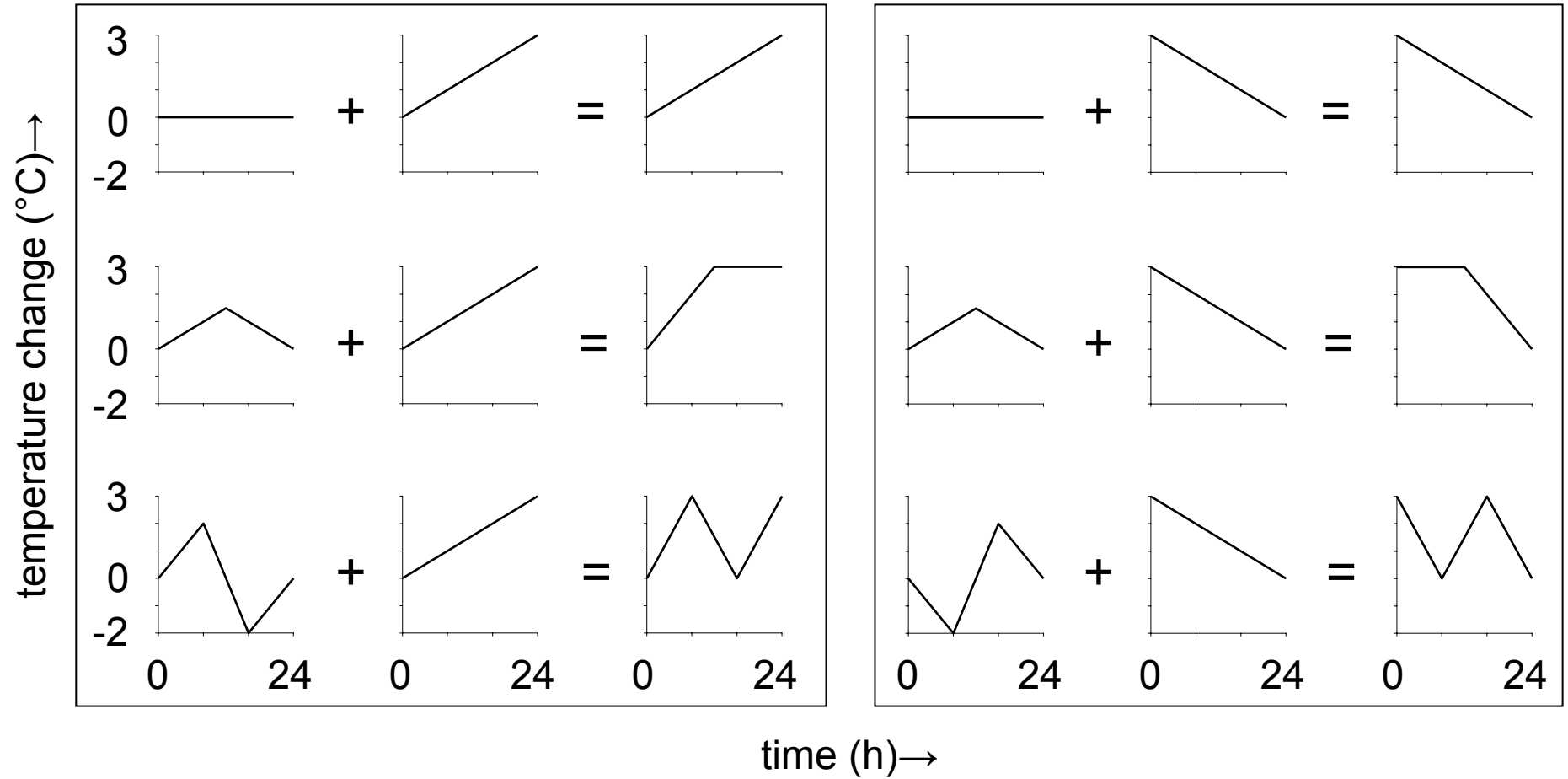

Supplement: Additional file 5 — Figure S4. Composite temperature profiles combining both daily temperature gradients and continuous increases or decreases. The left and right panels illustrate how the composite temperature profiles used for the experiments in Figure 7 resulted from combinations of daily temperature gradients with gradual 3°C temperature increases or decreases, respectively. The profiles in the first row represent combinations with constant temperature, whereas the profiles in the second and third rows involved daily temperature gradients with a 1.5°C or 4°C range, respectively. [file 1741-7007-7-49-S5.pdf]

activity  $\rightarrow$

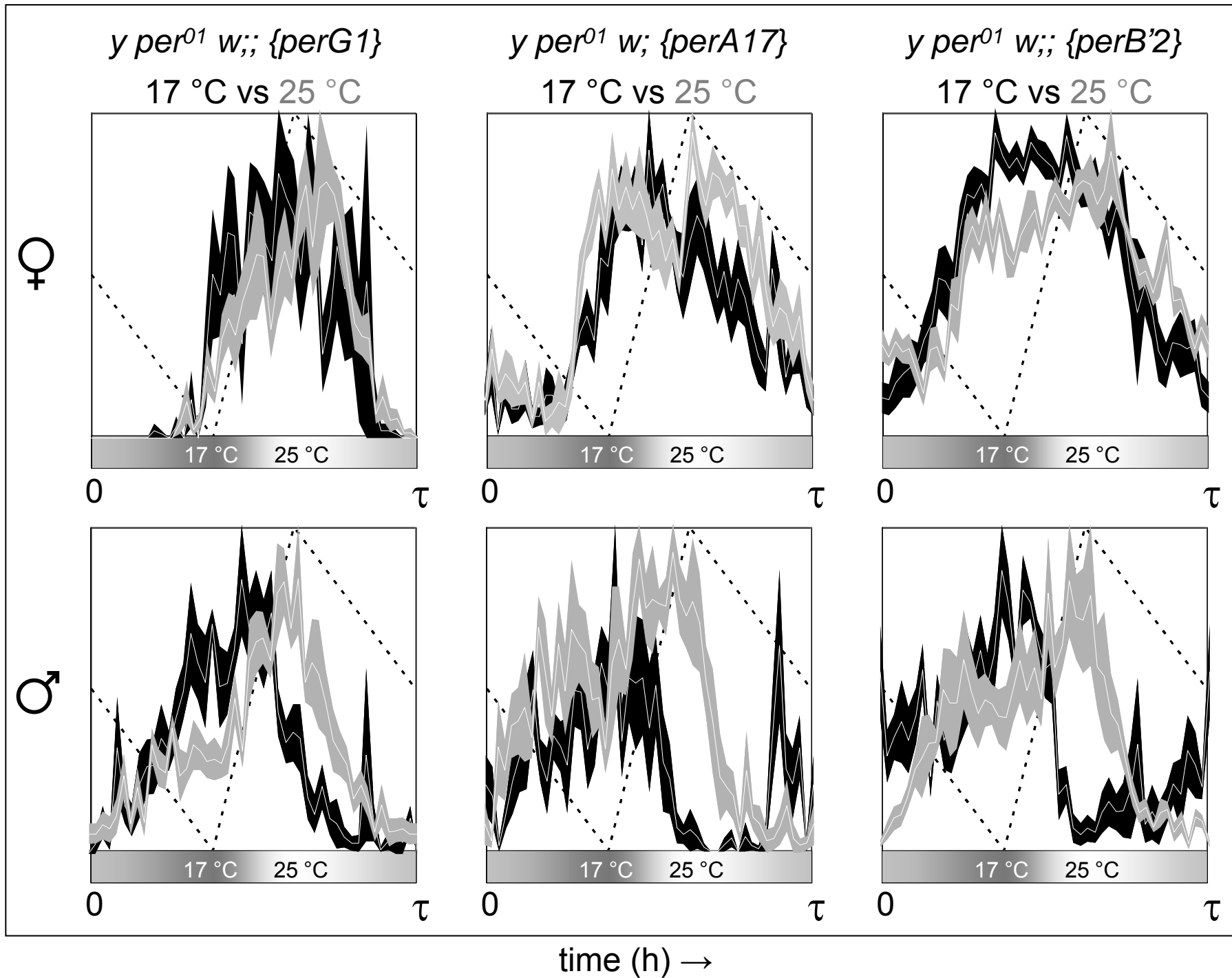

Supplement: Additional file 6 — Figure S5. The phase of temperature-entrained circadian behavior is modulated by the relative temperature of release into free run in the absence of thermosensitive splicing of the terminal intron of the per gene. The introduction of mutations that block temperature-dependent splicing of per did not prevent temperature-dependent modulation of the circadian phase of locomotor activity in male flies. Female circadian behavior appeared to be largely insensitive to the relative temperature during free running conditions, but this appeared to be due to the genotype used for transgenic rescue of the per01 mutation and unrelated to thermosensitive splicing of per. Temperature-dependent circadian locomotor behavior was determined for flies in which the per01 mutation was rescued by transgenes containing either wild-type genomic sequence (perG) or modified alleles that lack (perB') or constitutively maintain the terminal intron (perA), such that splicing at this intron was blocked. Following entrainment to a daily temperature gradient (ranging between 17°C and 25°C in constant dark (DD) conditions) flies were released under free running conditions at constant peak (25°C) or trough temperature (17°C) conditions. The diagrams represent median locomotor activity profiles relative to the intrinsic circadian period, tau. The dotted lines inside as well as the shading in the horizontal bars below the activity profiles represent the previous daily temperature gradient. [file 1741-7007-7-49-S6.pdf]
